# Molecular identification of *Cryptosporidium*, *Giardia*, and *Blastocystis* from stray and household cats and cat owners in Tehran, Iran

**DOI:** 10.1038/s41598-023-28768-w

**Published:** 2023-01-27

**Authors:** Poorya Karimi, Soheila Shafaghi-Sisi, Ahmad Reza Meamar, Elham Razmjou

**Affiliations:** Department of Parasitology and Mycology, School of Medicine, Iran University of Medical Sciences, Tehran, Iran

**Keywords:** Microbiology, Molecular biology

## Abstract

Cryptosporidiosis, giardiasis, and blastocystosis are among the most important parasitic diseases common between humans and cats. In addition, there are concerns about the possible transmission of zoonotic parasites from infected cats to humans. Hence, we investigated the molecular epidemiology of *Cryptosporidium* spp., *Giardia duodenalis*, and *Blastocystis* sp. in stray and household cats and cat owners. Our study was performed on 132, 33, and 33 fecal samples of stray and household cats, as well as cat owners in Tehran, Iran. *Cryptosporidium* spp. was identified using a nested PCR targeting the small subunit ribosomal RNA gene (*SSU* rRNA) and sequencing the internal amplified fragments. Furthermore, to perform multilocus genotyping of *G. duodenalis*, the ß-giardin (*bg*), glutamate dehydrogenase (*gdh*), and triosephosphate isomerase (*tpi*) genes were amplified to assess the DNA of *G. duodenalis* in the fecal samples of cats and cat owners. In addition, *Blastocystis* was detected by targeting the *SSU* rRNA gene, and the subtypes of *Blastocystis* were determined via the sequencing of amplicons. *Cryptosporidium felis* and *Cryptosporidium canis* were detected in seven stray cats (5.3%) and one household cat (3%). The *bg* gene of *G. duodenalis* was amplified and successfully sequenced in two (1.5%) stray cats and revealed assemblages F and B of *G. duodenalis*. Sequencing and phylogenic analysis of *SSU* rRNA gene nucleotide sequences of *Blastocystis* detected ST5 and ST10 in stray cats (1.5%), ST1 in household cats (9.1%), and ST1, ST2, ST3, and ST7 in owners (30.3%). The low prevalence of *Cryptosporidium, Giardia* and *Blastocystis* in cats and the presence of species/assemblages/subtypes with limited zoonotic potential indicate that cats had a minor role in their owners' infection in the investigated population. However, the presence of zoonotic protozoa in cats suggests the necessity of special attention to high-risk individuals during close contact with cats. Therefore, it is recommended that veterinarians, physicians, and urban managers plan to prevent, control, or treat these parasites to help the urban community live healthily alongside cats.

## Introduction

Zoonosis is any disease or infection naturally transmissible from vertebrate animals to humans^1^. Some of the most critical zoonosis infectious diseases are parasitic diseases transmitted to humans from companion and pet animals^1^. As the most popular pet, cats (*Felis catus*) have a close relationship with human societies. Even stray cats, which receive no standard veterinary care, freely pass in our yards and share public places with us, so they probably have a crucial role in transmitting parasitic diseases to humans. Cryptosporidiosis, giardiasis, and blastocystosis are among the most important parasitic diseases common between humans and cats^2^.

*Giardia* and *Cryptosporidium* can cause gastrointestinal disorders globally in many mammalian hosts with a wide range of clinical symptoms from self-limiting and asymptomatic to acute and life-threatening forms. The cysts/oocysts of these two enteric protozoan parasites are shedding in the hosts' feces. Accordingly, the main route of infection is fecal–oral transmission through contaminated food, water, or direct contact with infected humans or animals^3, 4^. Therefore, animals have an essential role in transmitting these parasites and the epidemiology of cryptosporidiosis and giardiasis^4^.

*Cryptosporidium*, an important apicomplexan parasite, comprises 44 species and more than 120 genotypes. Up to now, at least 19 species and four genotypes of *Cryptosporidium* have been reported in humans. However, most human cryptosporidiosis results from infection with *C. hominis*, *C. parvum*, *C. meleagridis*, *C. canis*, *C. felis*, and *C. ubiquitum*^3^. Cats are usually infected with *C. felis* with varying infection rates from 0 to 30% throughout the world, whereas in most reporting, infection rates were lower than 10%^5^. In addition, the zoonotic transmission of *C. felis* has been reported in numerous studies through close contact with cats in immunocompromised and immunocompetent humans^5^.

*Giardia duodenalis* infects a wide range of mammals, including humans. It is estimated that more than 280 million human giardiasis cases occur annually worldwide^6^. Hence, genotyping of *G. duodenalis* is a valuable tool for epidemiological studies, which are mainly performed using sequence analysis of PCR products from β-giardin (*bg*), triosephosphate isomerase (*tpi*), and glutamate dehydrogenase (*gdh*) genes. Over the last few decades, molecular studies have shown that *G. duodenalis* has eight genetically distinguishable assemblages (A-H) identified in mammalian hosts. Assemblages A and B have a broad range of human and animal hosts. Human infection mainly occurs with assemblages A and B, whereas assemblages C to H are more host-adapted, except for some assemblages C, D, E, and F in humans^6, 7^. The *G. duodenalis* infection of cats has been reported from 1.3 to 27.3%, mainly with the feline-specific assemblages F, with fewer reports of assemblages A, C, B, and D^6^.

*Blastocystis* is a common intestinal single-celled stramenopile protist with a vast range of hosts, from humans to insects^8^. Human *Blastocystis* infection has been reported from 0.5 to 100%^9^ globally and from 5 to 50% in Asia^10^. However, the pathogenicity and the public health importance of *Blastocystis* remain controversial, as it has been highly represented in asymptomatic healthy individuals as well as in a variety of acute or chronic gastrointestinal patients^11, 12^. *Blastocystis* is a polymorphic microscopic organism with high genetic diversity, especially across the *SSU* rRNA gene, which is the base of the classification of this genus to subtypes. The *Blastocystis* genus is currently classified into 28 subtypes (STs), which are ST1–ST17, ST21, ST23–ST29, and ST30–ST32^9, 11–14^. Although ST1–10, ST12, ST14, and ST16 have been reported from humans, more than 90% of *Blastocystis* STs found in humans are ST1–ST3 globally and ST4 mainly in Europe, whereas STs 5–9 sporadically, and STs 10, 12, 14, and 16 rarely have been reported from humans. However, all subtypes have been identified in animals except ST9, which has been reported up to now just in humans^9, 12^. Therefore, most *Blastocystis* STs have low host specificity, making zoonotic transmission possible^10^. The prevalence of *Blastocystis* infection in cats has been vastly reported from 0.0 to 100%. The reported subtypes in cats are ST1, ST3, ST4, ST10, and ST14^10, 15^.

Cats are mainly infected with intestinal parasites such as *Cryptosporidium* spp., *G. duodenalis*, and *Blastocystis* sp. Due to the close contact between humans and cats, there are concerns about the possible transmission of the zoonotic species/assemblages/subtypes of these parasites from infected cats to humans. Although the information on the regional prevalence of cat intestinal parasites is vital, few studies have been conducted on their prevalence and relation to human contamination in Iran. This information is necessary for cooperation between local veterinarians and public health authorities to develop effective strategies for treating and controlling parasites and educating pet owners^16^. Hence, this study was performed to identify and determine the molecular epidemiology of these zoonotic parasites in stray/household cats and cat owners of Tehran, the capital of Iran.

## Materials and methods

### Ethical approval

All cat owners who participated in this study gave informed written consent. The protocols of this study were reviewed by the Ethics Committee of Iran University of Medical Sciences and approved under the code IR.IUMS.FMD.REC 1396.31834. All methods were performed in accordance with the animal and human research guidelines and regulations from the Iranian Ministry of Health, Treatment, and Medical Education.

### Sample collection and preparation

The DNA of fecal samples of 132 and 33 stray and household cats previously collected^17^ were included in this study, along with 33 fecal samples collected from cat owners from January to September 2019, simultaneously collecting the fecal samples of their cats in Tehran (35.6892° N, 51.3890° E), Iran. In addition, demographic data concerning the age, sex, breed, weight, and residence area of cats, as well as the age, sex, and residence area of cat owners, were recorded. Cat owners reported no particular clinical symptoms at the sampling time in themselves or their cats. First, the sucrose flotation procedure was performed on 33 human fecal samples for concentrating oocysts/cysts of *Cryptosporidium*/*Giardia*^17, 18^. Then, the DNA of samples was extracted using the QIAamp DNA Mini Kit following the manufacturer’s instructions. The extracted DNA was stored at − 20 °C until further molecular analysis.

### Molecular identification, sequencing, and phylogenetic analysis

Molecular identification and characterization of *Cryptosporidium* spp. were performed by evaluating *Cryptosporidium* DNA in the fecal samples using a nested PCR targeting a 611-bp fragment of the small subunit ribosomal RNA (*SSU* rRNA) gene by specific primers designed by Silva et al.^19^ (Table 1). The species of *Cryptosporidium* isolates were determined by sequencing the internal amplified fragments.

**Table 1:** Genetic markers, Primers, and PCR amplification schemes for detecting and characterizing *Cryptosporidium spp., Giardia duodenalis,* and *Blastocystis sp.* in the fecal samples of cats and cat owners in Tehran, Iran.

Species	Gene	Primer nucleotide Sequences (5′–3′)	Amplicon size (bp)	Refs.	Cycling conditions	Refs.
*Cryptosporidium*	*SSU* rRNA	F: ACCTATCAGCTTTAGACGGTAGGGTAT R: TTCTCATAAGGTGCTGAAGGAGTAAGG F: ACAGGGAGGTAGTGACAAGAAATAACA R: AAGGAGTAAGGAACAACCTCCA	611	^19^	PCR 1: 45 s/94 °C, 45 s/56 °C, 45 s/72 °C, 39 cycles PCR2: 45 s/94 °C, 45 s/58 °C, 30 s/70 °C, 35 cycles	This study
*G. duodenalis*	*bg*	F: AAGCCCGACGACCTCACCCGCAGTGC R: GAGGCCGCCCTGGATCTTCGAGACGAC F: GAACGAACGAGATCGAGGTCCG R: CTCGACGAGCTTCGTGTT	511	^20^	PCR 1: 30 s/95 °C, 30 s/65 °C, 60 s/72 °C, 35 cycles PCR 2: 30 s/95 °C, 30 s/55 °C, 60 s/72 °C, 35 cycles	^23^
*G. duodenalis*	*gdh*	F: TCAACGTYAAYCGYGGYTTCCGT R: GTTRTCCTTGCACATCTCC F: CAGTACAACTCYGCTCTCGG R: GTTRTCCTTGCACATCTCC	430	^21^	PCR 1: 2 min/94 °C, 60 s/61 °C, 2 min/68 °C, 1 cycle; 30 s/94 °C, 20 s/61 °C, 20 s/68 °C, 30 cycles PCR 2: 2 min/94 °C, 60 s/60 °C, 2 min/65 °C, 1 cycle; 30 s/94 °C, 20 s/60 °C, 20 s/65 °C, 15 cycles	^18^
*G. duodenalis*	*tpi*	F: AAATIATGCCTGCTCGTCG R: CAAACCTTITCCGCAAACC F: CCCTTCATCGGIGGTAACTT R: GTGGCCACCACICCCGTGCC	530	^22^	PCR 1: 45 s/94 °C, 45 s/50 °C, 60 s/72 °C, 35 cycles PCR 2: 45 s/94 °C, 45 s/58 °C, 60 s/72 °C, 35 cycles	^23^
*Blastocystis*	*SSU* rRNA	F: GGAGGTAGTGACAATAAATC R: TAAGACTACGAGGGTATCTA	550–585	^24^	60 s/94 °C, 45 s/56 °C, 45 s/72 °C, 35 cycles	^24^

Molecular identification and multilocus genotyping of *G. duodenalis* were performed by amplification of the ß-giardin (*bg*), glutamate dehydrogenase (*gdh*), and triosephosphate isomerase (*tpi*) genes to assess the DNA of *G. duodenalis* in the fecal samples of cats and cat owners. For this purpose, a 511-bp fragment of the *bg* gene^20^, a 432-bp fragment of the *gdh* gene^21^, and a 530-bp fragment of the *tpi* gene^22^ were amplified by a nested PCR, a semi-nested PCR, and a nested PCR, respectively, according to previous procedures^18, 23^ (Table 1). The assemblages, sub-assemblages, and genotypes of *G. duodenalis* isolates were identified by sequencing each marker's internal amplified fragments.

Molecular identification and characterization of *Blastocystis* were accomplished by a PCR method targeting a 550- to a 585-bp nucleotide fragment of the *SSU* RNA gene on the genomic DNA extracted directly from fecal samples^24^. In addition, the subtypes of isolates were identified via the sequencing of amplicons.

All PCRs were performed in 25 µL of the amplification reaction mixture using primer pairs and conditions listed in Table 1. The amplification reaction mixture consisted of 12.5 µL of 2 × Taq DNA Polymerase Master Mix RED (Amplicon III, Denmark, Copenhagen, cat. no. 180301), 2.5 µL of primer pair mix (0.4 µM of each primer in the primary and secondary PCR reactions for detection of *Cryptosporidium* spp.; 0.2 μM for *tpi* and *bg* loci or 0.5 µM for *gdh* locus in the PCR reactions for detection of *G. duodenalis*, or 0.5 µM for detection of *Blastocystis*), and 2 μM of template DNA of each sample in the single PCR reaction or the primary PCR reaction of nested or semi-nested PCR reaction and 2 μM of PCR products in the secondary PCR reaction of nested or semi-nested PCR. Amplicons were analyzed on 1.5% (w/v) agarose gel. In the case of positive samples, the PCR or the secondary PCR reaction of nested or semi-nested PCR was repeated in 50 µL of the amplification reaction mixture with each parasite’s corresponding primer pairs. After electrophoresis on 1% agarose gels, the resulting amplicons were purified with the MinElute Gel Extraction Kit (Qiagen, Hilden, Germany) for sequencing in both directions using forward and reverse primers (Macrogen Inc., Seoul, South Korea). The results of each parasite sample's corresponding forward and reverse sequences were read and verified by the Chromas software (Technelysium Pty Ltd., Queensland, Australia) and subsequently aligned and assembled using MEGA X software (www.megasoftware.net). The species of *Cryptosporidium*, the assemblage, sub-assemblage, and genotype of *G. duodenalis*, as well as the subtype of *Blastocystis* sp., were identified by comparing the homology of the final nucleotide sequences with corresponding sequences retrieved from the GenBank database (http://blast.ncbi.nlm.nih.gov). The achieved nucleotide sequences were deposited in the GenBank under accession numbers LC700089–LC700096 for the *SSU* rRNA gene of *C. felis* and *C. cains,* LC700097–LC700098 for the *bg* gene of *G. duodenalis,* and LC700104–LC700118 for the *SSU* rRNA gene of *Blastocystis*. The phylogenetic analysis was accomplished in MEGA X (www.megasoftware.net) using the maximum likelihood (ML) algorithm with evolutionary distances calculated by the Kimura-2 parameter (K2) model and a bootstrap value of 1000 to estimate the consistency of clusters.

### Statistical analyses

Statistical analysis was conducted using SPSS 24.0 Statistical Software (SPSS Inc., Chicago, IL, USA). Chi-square tests estimated potential associations between qualitative variables with a 95% confidence interval (CIs), and the *p* value of < 0.05 were considered significantly different.

## Results

### Prevalence of *Cryptosporidium*, *Giardia*, and *Blastocystis* among stray/household cats and their owners

The prevalence of *Cryptosporidium* spp. was 5.3% (7/132; 95% CI 2.6–10.5), 3.0% (1/33; 95% CI 0.5–15.3), and 0.0% (0/33; 95% CI 0.0–10.4) in stray cats, household cats, and cat owners, respectively. The *SSU* rRNA gene nucleotide of *Cryptosporidium* spp. was not found in the feces of cat owners. Statistical differences were not observed between the prevalence of *Cryptosporidium* spp. in stray cats, household cats, and cat owners. The prevalence of *Cryptosporidium* spp. infection based on age, sex, breed, weight, and the urban region in stray and household cats is shown in Tables 2 and 3. *Cryptosporidium* spp. infection was not statistically related to age, breed, weight, and urban region of stray or household cats. However, the prevalence of *Cryptosporidium* spp. in stray cats was affected by sex (*p* = 0.028), as it was in female cats (16.7%, 6/60; 95% CI 4.7–20.1) higher than in male cats (1.4%, 1/72; 95% CI 0.2–7.5). The only *Cryptosporidium*-infected household cat was a two-year male Persian cat with a 3.8 kg weight.

**Table 2:** The demographics and the prevalence of *Cryptosporidium* spp., *Giardia duodenalis*, and *Blastocystis* sp. in the stray cats of Tehran, Iran.

Factors	Samples % (N)	*Cryptosporidium* spp.		*Giardia duodenalis*		*Blastocystis* sp.	
		% (N)	*p*	% (N)	*p*	% (N)	*p*
Age							
< 1 year	47.0% (62)	3.2% (2)	0.316	1.6% (1)	0.931	1.6% (1)	0.467
> 1 year	53.0% (70)	7.1% (5)		1.4% (1)		1.4% (1)	
Sex							
Male	54.5% (72)	1.4% (1)	0.028	1.4% (1)	0.896	2.8% (2)	0.193
Female	45.5% (60)	10.0% (6)		1.7% (1)		0.0% (0)	
Breed							
DSH	72.7% (96)	6.2% (6)	0.719	2.1% (2)	0.683	0.0% (0)	0.054
DLH	25.8% (34)	2.9% (1)		0.0% (0)		5.9% (2)	
Persian	1.5% (2)	0.0% (0)		0.0% (0)		0.0% (0)	
Weight							
< 2 kg	47.0% (62)	4.8% (3)	0.470	1.6% (1)	0.854	1.6% (1)	0.854
2–4	40.2% (53)	7.5% (4)		0.8% (1)		1.9% (1)	
> 4 kg	12.8% (17)	0.0% (0)		0.0% (0)		0.0% (0)	
Urban region							
North	28.8% (38)	5.3% (2)	0.794	0.0% (0)	0.430	0.0% (0)	0.008
Center	18.9% (25)	4.0% (1)		0.0% (0)		0.0% (0)	
South	12.9% (17)	11.8% (2)		5.9% (1)		0.0% (0)	
West	26.5% (35)	2.9% (1)		2.9% (1)		0.0% (0)	
East	12.9% (17)	5.9% (1)		0.0% (0)		11.8% (2)	
Total	100.0% (132)	5.3% (7)		1.5% (2)		1.5% (2)

**Table 3:** The demographics and the prevalence of *Cryptosporidium* spp. and *Blastocystis* sp. in the household cats of Tehran, Iran.

Factors	Samples % (N)	*Cryptosporidium* spp.		*Blastocystis* sp.	
		% (N)	*p*	% (N)	*p*
Age					
< 1 year	15.2% (5)	0.0% (0)	0.688	20.0% (1)	0.357
>1 year	84.8% (28)	3.6% (1)		7.1% (2)	
Sex					
Male	51.5% (17)	5.9% (1)	0.325	5.9% (1)	0.509
Female	48.5% (16)	0.0% (0)		12.5% (2)	
Breed					
DSH	33.3% (11)	0.0% (0)	0.684	9.1% (1)	0.291
DLH	9.1% (3)	0.0% (0)		33.3% (1)	
Persian	57.6% (19)	5.3% (1)		5.3% (1)	
Weight					
< 2 kg	8.2% (6)	0.0% (0)	0.684	16.7% (1)	0.649
2–4	57.6% (19)	5.3% (1)		11.1% (1)	
> 4 kg	24.2% (8)	0.0% (0)		12.5% (1)	
Urban region					
North	21.2% (7)	0.0% (0)	0.811	0.0% (0)	0.024
Center	24.2% (8)	0.0% (0)		12.5% (1)	
South	3.0% (1)	0.0% (0)		100% (1)	
West	39.4% (13)	7.7% (1)		7.7% (1)	
East	12.1% (4)	0.0% (0)		0.0% (0)	
Total	100.0% (33)	3.0% (1)		9.1% (3)	

The prevalence of *G. duodenalis* was 1.5% (2/132; 95% CI 0.4–5.4), 0.0% (0/33; 95% CI 0.0–10.4), and 0.0% (0/33; 95% CI 0.0–10.4) in stray cats, household cats, and cat owners, respectively. The DNA of *G. duodenalis* was not found in the feces of household cats or cat owners. No statistical differences were observed in the prevalence of *G. duodenalis* infection in stray cats, household cats, and cat owners, as well as between any demographic variables and infection with *G. duodenalis* in stray cats. The association between *G. duodenalis* infection and stray cats' demographic variables is revealed in Table 2.

The prevalence of *Blastocystis* sp. was 1.5% (2/132; 95% CI 0.4–5.4), 9.1% (3/33; 95% CI 3.1–23.6), and 30.3% (10/33; 95% CI 17.4–47.3) in stray cats, household cats, and cat owners, respectively. No statistical differences were found between the prevalence of *Blastocystis* sp. infection in stray and household cats, as well as between household cats and cat owners. However, the prevalence of *Blastocystis* sp. infection in cat owners was statistically higher than in stray cats. The prevalence of *Blastocystis* sp. infection associated with demographic variables of stray cats, household cats, and cat owners are shown in Tables 2, 3, and 4, respectively. There was no statistically significant relationship between age, sex, breed, weight, and infection with *Blastocystis* in stray and household cats. The Chi-Square statistical analysis showed a significant relationship between the prevalence of *Blastocystis* sp. and the urban region in the stray cats (*p* = 0.008) and household cats (*p* = 0.024). The highest prevalence of *Blastocystis* infection was detected in stray cats (2/17; 11.8%; 95% CI 3.3–34.3) and household cats (1/1; 100.0%; 95% CI 20.7–100.0) living in the east and south of Tehran, respectively. However, there was no statistically significant relationship between any demographic variables and infection with *Blastocystis* in the cat owners.

**Table 4:** The demographics and the prevalence of *Blastocystis* sp. in the cat owners of Tehran, Iran.

Factors	Samples % (N)	*Blastocystis* sp.	
		% (N)	*p*
Age			
20–29	27.3% (9)	11.1% (1)	0.051
30–39	33.3% (11)	45.5% (5)	
40–49	18.2% (6)	0.0% (0)	
> 50	21.2% (7)	57.1% (4)	
Sex			
Male	57.6% (19)	36.9% (7)	0.341
Female	42.4% (14)	21.4% (3)	
Urban region			
North	21.2% (7)	14.3% (1)	0.354
Center	24.2% (8)	37.5% (3)	
South	3.0% (1)	100.0% (1)	
West	39.4% (13)	23.1% (3)	
East	12.1% (4)	50.0% (2)	
Total	100.0% (33)	30.3% (10)	

Totally, molecular analysis revealed infection with *Cryptosporidium* spp.*, G. duodenalis,* or *Blastocystis* sp. occurred in 11 of 132 (8.3%; 95% CI: 4.7–14.3%) stray cats, although *Cryptosporidium* spp. or *Blastocystis* sp. was detected in 4 of 33 (12.1%; 95% CI: 4.3–27.3%) household cats. However, mixed infections of these parasites were not observed in any of the stray/household cats or their owners.

### *Cryptosporidium* species/genotype in stray and household cats

**Figure 1:**
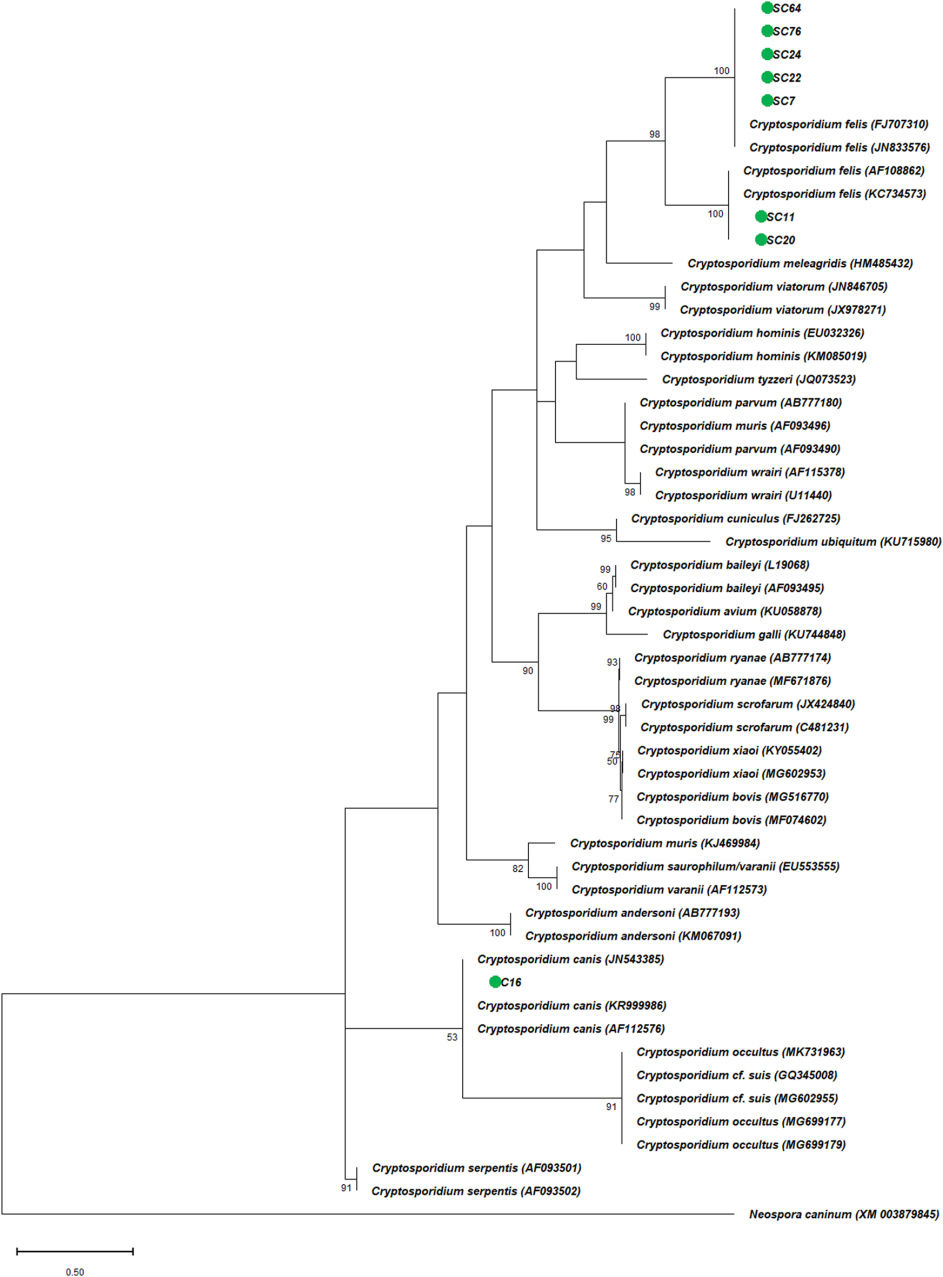
The phylogram of *Cryptosporidium* spp. was inferred based on the nucleotide sequences of *SSU* rRNA gene. The evolutionary relationship of *Cryptosporidium* spp. was constructed by the Maximum Likelihood method and Kimura 2-parameter model, based on the nucleotide sequences of *SSU* rRNA gene of *C. felis* and *C. canis* isolated from stray cats [SC] and household cats [C] in this study (green circles) compared with nucleotide sequences of *Cryptosporidium* species retrieved from GenBank, with *Neospora caninum* (XM_003879845) as outgroup. Bootstrap values obtained from 1000 replicates are indicated on branches in percentage; only bootstrap values > 50% are displayed. Evolutionary analyses were conducted in MEGA X.

Sequencing of the *SSU* rRNA gene identified two *Cryptosporidium* species among the eight *Cryptosporidium*-positive samples, including seven *C. felis* in stray cats and one *C. canis* in household cats. In addition, the multiple alignments of the *SSU* rRNA gene nucleotide fragment sequences of seven *C. felis* samples with the deposited sequences retrieved from GenBank showed two nucleotide patterns. Five *C. felis* isolates had 100% homology with GenBank sequence accession numbers FJ707310 and JN833576, and the other two isolates revealed 100% sequence identity to GenBank sequence accession numbers AF108862 and KC734573 (Supplementary Table 1). Sequencing analysis of the only *C. canis* isolate showed 100% homology with nucleotide deposited sequence in GenBank (AF112576, KR999986, and JN543385). Furthermore, the phylogenetic trees of the *SSU* rRNA gene nucleotide fragment sequences of the *Cryptosporidium* parasites isolated in this study compared with nucleotide sequences of *Cryptosporidium* species retrieved from GenBank confirmed the sequencing analysis observations (Fig. 1).

### *Giardia duodenalis* assemblages and genotypes in stray cats

The PCR amplification of the *bg, gdh*, and *tpi* genes showed two positive samples of *G. duodenalis* in stray cats, which were detected only at the *bg* locus. No DNA amplification was performed at the *gdh* or *tpi* loci. The sequencing analysis of two *bg*-positive samples revealed two assemblages, B (50.0%) and F (50.0%).

**Figure 2:**
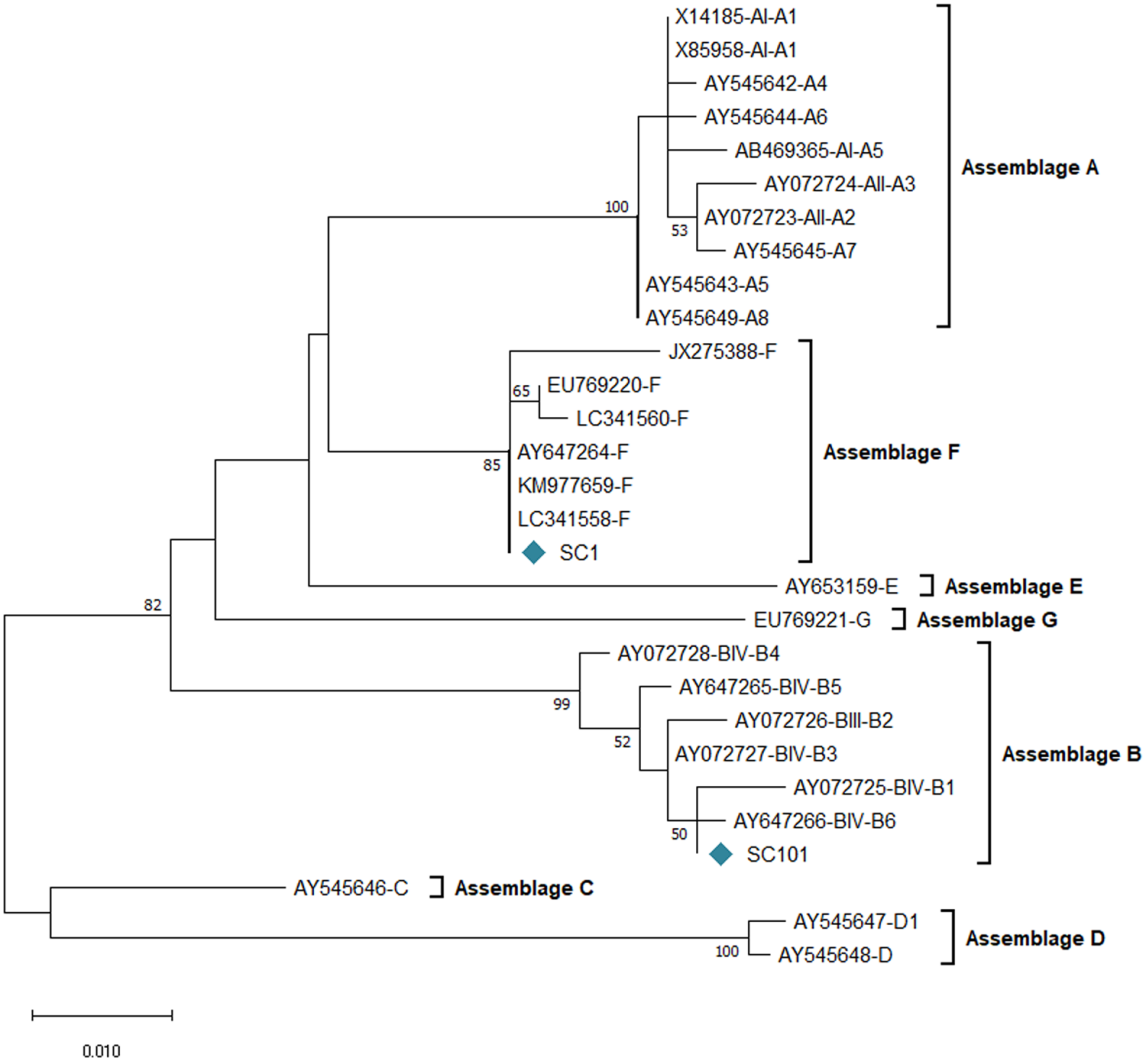
The phylogram of *Giardia duodenalis* was inferred based on the nucleotide sequences of the β-giardin (*bg*) gene. The evolutionary relationship of *G. duodenalis* was constructed by the Maximum Likelihood method and Kimura 2-parameter model, based on the nucleotide sequences of the *bg* gene of *G. duodenalis* isolated from stray cats [SC] in this study (light sea green rhombus) compared with nucleotide sequences of known assemblages retrieved from GenBank. Bootstrap values obtained from 1000 replicates are indicated on branches in percentage; only bootstrap values > 50% are displayed. Evolutionary analyses were conducted in MEGA X.

Sequence multiple alignment analysis of the *bg* gene with reference sequences classified the assemblage B isolate at the BIV sub-assemblage. In addition, it revealed some single nucleotide polymorphisms (SNPs), resulting in a novel genotype. The comparative analysis, with the reference sequences of B1–B6 genotypes, indicated the occurrence of two SNPs where a thymidine substituted a cytosine at the position of 185 (T185C) or 210 (T210C) compared with genotype B6 (AY647266), or B3 (AY072727), respectively (Supplementary Table 2). However, no SNPs were detected in the assemblage F isolate compared with the reference sequence (AY647264) representing F assemblage. The *bg* gene phylogenetic analysis classified assemblage F isolate (SC1) in one cluster with assemblage F demonstrating 100% homology with reference sequences (AY647264, KM977659, LC341558), and assemblage B isolate (SC101) in one group with sub-assemblage BIV, among genotype B3 and B6 sequence references (Fig. 2).

### *Blastocystis* subtypes and intra-subtype variability in stray cats, household cats, and cat owners

**Figure 3:**
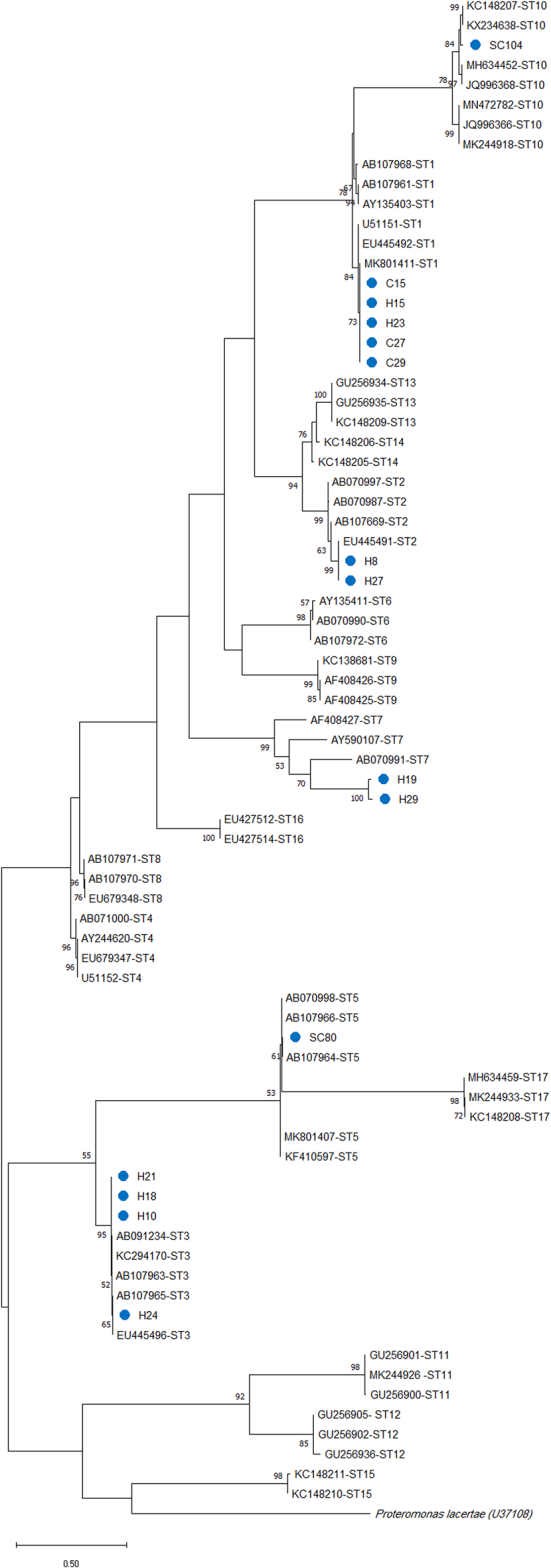
The phylogram of *Blastocystis* subtypes was inferred based on the nucleotide sequences of *SSU* rRNA gene. The evolutionary relationship of *Blastocystis* subtypes was constructed by the Maximum Likelihood method and Kimura 2-parameter model, based on the nucleotide sequences of *SSU* rRNA gene of *Blastocystis* isolated from stray cats [SC], household cats [C], and cat owners [H] in this study (blue circles) compared with nucleotide sequences of *Blastocystis* subtypes retrieved from GenBank, with *Proteromonas lacertae* (U37108) as outgroup. Bootstrap values obtained from 1000 replicates are indicated on branches in percentage; only bootstrap values > 50% are displayed. Evolutionary analyses were conducted in MEGA X.

Sequencing and phylogenetic analysis of the amplified fragment of the *SSU* rRNA gene recognized six subtypes of *Blastocystis*, including ST5 and ST10 in stray cats, ST1 in household cats, and ST1, ST2, ST3, and ST7 in cat owners. The multiple alignments of the *SSU* rRNA gene nucleotide fragment sequences of five ST1 isolates with the deposited sequences retrieved from GenBank showed one nucleotide pattern, which had three SNPs with GenBank sequence accession number U51151, where an adenine substituted guanine at the position of 172 (A172G), and thymidine substituted cytosine at the position of 195 (T195C) and 247 (T247C). In contrast, this ST1 nucleotide pattern revealed one SNP at the position of 260 (C260G) compared with MK801411 (Supplementary Table 3). Sequencing analysis of two ST2 isolates had one pattern with one SNP (T15A) compared with EU445491. Four ST3 isolates from humans revealed two different patterns. One isolate showed 100% homology with nucleotide deposited sequences in GenBank (AB107965 and EU445496), and the other three isolates revealed one SNP (G164A) compared with nucleotide sequences retrieved from GenBank (AB107963 and KC294170). Sequencing analysis of the only ST5 isolate from the stray cat showed one SNP compared with nucleotide sequences isolated from pig AB107964 (G385A) and cattle AB107966 (A185T). Two ST7 isolates of humans revealed two different patterns, with the highest nucleotide diversity (30–31 SNPs) compared with nucleotide sequences retrieved from GenBank (AF408427 and AB070991). Finally, the only ST10 isolate from the stray cat showed 12 SNPs compared with the nucleotide sequence isolated from the camel (KC148207). The phylogenetic analysis of the *SSU* rRNA gene nucleotide fragment sequences of the *Blastocystis* subtypes isolated in this study compared with nucleotide sequences of *Blastocystis* subtypes retrieved from GenBank confirmed the sequencing analysis observations (Fig. 3).

## Discussion

In the present study, molecular analyses were performed to identify species, assemblages, or subtypes of *Cryptosporidium* spp., *G. duodenalis*, and *Blastocystis* sp. in stray/household cats and their owners in Tehran to acquire sufficient information about the prevalence of these intestinal parasites in stray/household cats and to assess the potential role of household cats in the transmission of these zoonotic parasitic infections to their owners.

Consistent with the global prevalence of *Cryptosporidium* infection (4.2%) reported in cats by molecular diagnostic methods^25^, we found *Cryptosporidium* spp. infection in 4.8% (8/165; 95% CI 2.6–10.5) of the investigated cats. Although the prevalence of *Cryptosporidium* spp. in the investigated stray cats (5.3%) was in the ranges reported in China (5.6–5.8%)^26^, it is lower than previous similar studies done in Australia (13.4%)^27^ and the Czech Republic (7.4%)^2^. In contrast, it is higher than in Italy (0%)^28^ and South Korea (0.6%)^29^. However, the prevalence of *Cryptosporidium* spp. in household cats (3%) was lower than in molecular studies done in Australia (7.1%)^27^ and Japan (12.7%)^30^. In comparison, it is higher than in Italy (0%)^28^, China (0.6%)^26^, the Czech Republic (0.8%)^2^, Japan (2%)^31^, and the others study in Iran (0.7%)^32^. These discrepancies might result from the study populations, geographical distribution, different identification methods, and various levels of living conditions. As reported before^27^, high *Cryptosporidium* infection in the cats living in a refuge center might be related to their close contact and keeping conditions. The investigation of possible factors associated with *Cryptosporidium* infection revealed that it was more frequent in stray female cats (*p* = 0.028), which contradicted previous reports^2, 29^. There is no evidence that sex has a role in increasing the chance of infection with *Cryptosporidium* species. Therefore, more studies with a larger sample size are necessary to evaluate this relationship. Furthermore, the *Cryptosporidium* spp. infection was not detected in the cat owners, which could be attributed to good veterinary care and, considering hygiene principles, correspondingly no infection in their cats, except for one case. There are limited studies on the *Cryptosporidium* infection in household cats and their owners. There are merely two reports, one concomitant infection of a Swedish woman and her cat with *C. felis* and another detection of *C. felis* infection in a cat and its owner^33, 34^. Consistent with previous findings^2, 26, 27, 29^, *C. felis* was the only species identified in the *Cryptosporidium*-positive isolates from stray cats, whereas *C. canis* was distinguished in the sole infected household cat. Based on our knowledge, this is the first report of finding *C. canis* in household cats. This two-year male Persian cat was adopted at three months old from a pet shop, and about one month before sampling was in pet boarding for a week due to his owner's travel, where he might have been infected with *C. canis*. Further studies on the prevalence of this species in cats seem necessary. Although *C. felis* is the main species infecting cats, it is one of the six prevalent species infecting humans after *C. hominis* and *C. parvum*, which are responsible for 95% of human cryptosporidiosis^3, 4^. The zoonotic potential of feline and canine cryptosporidiosis concerns veterinarians and physicians worldwide due to the close contact between humans and companion animals^3, 5, 34^. However, the low infection rate of household cats (3%) and no infection found in the cat owners suggest a limited role of cats in the human cryptosporidiosis in the studied population. Nevertheless, special attention is necessary to the zoonotic potential of these species, especially in children and immunocompromised individuals who have close contact with cats.

The prevalence of *G. duodenalis* infection in stray cats (1.5%) was lower than in South Korea (3.8%), the Czech Republic (7.4%), and Italy (10.9%)^2, 28, 29^, whereas it was higher than in China (0%)^35^. However, we did not detect any *G. duodenalis* infection in household cats by amplifying *bg*, *tpi*, and *gdh* loci, which was less than in Shiraz, Iran (1.3%), China (1.2%), Italy (4%), the Czech Republic (5%), and Denmark (10.5%)^2, 26, 28, 32, 36^. The different ranges of *G. duodenalis* infection reported in the cats might reflect the various loci and molecular methods applied for the detection of DNA of this parasite. As seen in our study, the *tpi* and *gdh* loci could not detect the DNA of two positive samples detected by the *bg* locus, which is consistent with the different amplification rates of these three genetic loci^37^. Moreover, geographical distribution and studied populations affecting the living conditions have the leading role in the prevalence of *G. duodenalis* in cats^6^. Accordingly, consistent with the previous reports, we found higher infection rates in stray cats than in household cats^2, 27^. Furthermore, the assemblage F of *G. duodenalis* is mainly detected in cats, followed by assemblage A^6^. However, assemblage B has been reported in a few studies from China, Europe, and Australia^21, 37, 38^. Here, we found the zoonotic assemblage B for the first time in Iran and the feline-specific assemblage F in stray cats. Consistent with other reports from the world^6^, the further sequence alignment and phylogenetic analysis of two isolates showed that assemblage F had no SNP, while assemblage B revealed some SNPs. Identifying only two *G. duodenalis* infection cases in stray cats and no infection in the household cats or their owners, suggesting cats had a minimal potential role in human giardiasis in the studied population.

The epidemiology of *Blastocystis* in cats is controversial, like other biological aspects of this parasite. The molecular prevalence of *Blastocystis* in cats has been reported from 0.6 to 100%, whereas some studies reported no presence of *Blastocystis* in Carnivora, including cats^8, 15^. We found *Blastocystis* infection in 3% (5/165; 95% CI 1.3–6.9) of the investigated cats. The prevalence of *Blastocystis* in stray cats (1.5%) was lower than in previous studies in Fars Province, Iran (17.7%)^39^, Malaysia (20.0%)^40^, the USA (11.6%)^41^, and Turkey (3.6%)^42^ and higher than in South Korea (0.6%)^29^. At the same time, the prevalence of *Blastocystis* in household cats (9.1%) was lower than in molecular studies in Australia (100%)^43^ and Turkey (100.0%)^44^ and higher than in China (0.6%)^45^, the USA (0%)^41^, Thailand^46^, and Spain (0%)^47^. Although there have been some studies on the presence of *Blastocystis* in cats, this data is not enough to conclude a reason for explaining so much variety in the distribution of this parasite in different studies. Albeit, it is likely that the prevalence of *Blastocystis* attributed to the cats’ standard of care and hygiene. This relation has been revealed in a study in which *Blastocystis* infection was only reported in the shelter cats (11.6%), while owned cats were not infected^41^. Although in our study, the *Blastocystis* infection in household cats was more prevalent than in stray cats, which suggested that owned cats might get infected via close contact with their owner. Consistent with the prevalence of *Blastocystis* infection reported in Asia (5% to 50%) and healthy general populations of Iran (3.3% to 30.1%) by molecular diagnostic methods^10^, we found *Blastocystis* infection in 30.3% of the cat owners. The variety in the prevalence of *Blastocystis* from 0.5 to 100% across the world is considered related to inadequate hygiene and sanitation that increased the chance of potential anthroponotic and zoonotic transmission via the fecal–oral route contamination^9^. However, many studies have suggested that *Blastocystis* is one of the gastrointestinal microbiota of healthy individuals^9, 11, 12, 48^. Therefore, the latter hypothesis is more plausible about the human samples in our study. Furthermore, the phylogenetic analysis documented the ST5 and ST10 in stray cats, which are mainly reported from pigs and cattle worldwide, with less zoonotic importance^10, 15, 49^. Although, in the only published molecular study performed on stray cats in Fars Province, Iran, five *Blastocystis* subtypes were determined, ST1, ST3, ST4, ST10, and ST14, with the more zoonotic significance^39^. Moreover, the *Blastocystis* subtypes of ST1, ST3, and ST10 in the USA^41^, ST1 in Malaysia^40^, and ST4 in South Korea^29^ and Turkey^42^ have been reported from the shelter or stray cats. While the phylogenetic analysis distinguished a zoonotic *Blastocystis* subtype, ST1, in the three household cats, which is the subtype also reported in household cats in China^45^ and Australia^43^, another zoonotic *Blastocystis* subtype, ST3, was distinguished in three household cats in Turkey^44^. In addition, we identified the *Blastocystis* subtypes of ST1, ST2, ST3, and ST7, with a predominance of ST3, in cat owners, which is consistent with human-reported subtypes in Iran and Asia^10^. In only one case, both household cats and their owner were infected with *Blastocystis* ST1, suggesting a possible common source of infection. However, due to the low prevalence rates and nonspecific STs presented in stray or household cats, it seems cats were not the main potential reservoirs for transmitting human *Blastocystis* infection.

## Conclusion

The low prevalence of *Cryptosporidium* (4.8%) and *Giardia* (1.2%) in cats and the presence of species and assemblages with low zoonotic potential, as well as no infections found in cat owners, limit the role of cats in the human cryptosporidiosis or giardiasis in the investigated population. Comparing the prevalence of *Blastocystis* in cats (3%) and their owners (30.3%), in addition to nonspecific STs detected in stray cats, shows a minor role of cats in human infection with *Blastocystis*. However, the presence of zoonotic protozoa in cats needs special attention from cat enthusiasts, especially children and immunocompromised individuals. Therefore, it is recommended that veterinarians, physicians, and urban managers plan to prevent, control, or treat these parasites to help the urban community live healthily alongside these companion animals.

## Supplementary Information

Supplementary Information.

## Data Availability

All data generated or analyzed during this study are included in this published article and its supplementary information files. The sequences were submitted to DDBJ/EMBL/GenBank databases (https://www.ncbi.nlm.nih.gov/nuccore/) under accession numbers LC700089–LC700098 and LC700104–LC700118.

## Abbreviations

*bg*: ß-giardin
bp: Base pair
CI: Confidence interval
DLH: Domestic long hair
DSH: Domestic short hair
*gdh*: Glutamate dehydrogenase
K2: Kimura 2-parameter
ML: Maximum likelihood
SNPs: Single nucleotide polymorphisms
*SSU* rRNA gene: Small subunit ribosomal ribonucleic acid gene
ST: Subtype
*tpi*: Triosephosphate isomerase
