# Supplementary material for: Molecular identification of Cryptosporidium, Giardia, and Blastocystis from stray and household cats and cat owners in Tehran, Iran
Source: Sci Rep. 2023 Jan 27;13:1554. doi: 10.1038/s41598-023-28768-w (PMC9883249; doi:10.1038/s41598-023-28768-w)
Supplement: Supplementary file 1 — Supplementary Information. [file 41598_2023_28768_MOESM1_ESM.pdf]

Molecular identification of *Cryptosporidium*, *Giardia*, and *Blastocystis* from stray and household cats and cat owners in Tehran, Iran

Poorya Karimi[1], Soheila Shafaghi-Sisi[1], Ahmad Reza Meamar[1], and Elham Razmjou[1]*

[1] Department of Parasitology and Mycology, School of Medicine, Iran University of Medical Sciences, Tehran, Iran

Supplementary information

Supplementary Table 1. The multiple alignments nucleotide sequences of SSU rDNA fragment of *Cryptosporidium felis* isolated in this study with reference sequences retrieved from GenBank represent the position of intra- species substitutions in *Cryptosporidium felis*.

Isolate/access no	Nucleotide position	
	198	228
Cryptosporidium felis (AF108862)	T	T
Cryptosporidium felis (KC734573)	.	.
Cryptosporidium felis (FJ707310)	A	-
Cryptosporidium felis (JN833576)	A	-
SC11, SC20	.	.
SC7, SC22, SC24, SC64, SC76	A	-

Supplementary Table 2. Multiple alignments sequences of assemblage B reference sequences genotypes of *Giardia duodenalis* retrieved from GenBank, with assemblage B isolate in this study at the β -giardin (*bg*) locus.

Isolate/access no	Nucleotide position from the start of the gene										
	185	210	228	273	327	354	357	438	471	564	609
AY072725-BIV-B1	T	T	A	A	T	T	T	T	C	T	C
AY072726-BIII-B2	.	C	.	G	C	C	C	C	.	.	.
AY072727-BIV-B3	.	C	.	.	C	C	.	C	.	.	.
AY072728-BIV-B4	.	C	G	.	C	.	.	C	.	C	T
AY647265-BIV-B5	.	C	.	.	C	.	.	C	T	.	.
AY647266-BIV-B6	C	.	.	.	C	C	.	C	.	.	.
SC101	.	.	.	.	C	C	.	C	.	.	.

Numbers represent the nucleotide position of intra-genotypic substitutions in BIV and BIII sub-assemblages and genotypes [1, 2]. Dots indicate nucleotide identity to the sub-assemblages BIV, genotype B1 (AY072726) reference sequences.

References

1. Wielinga CM, Thompson RCA. Comparative evaluation of *Giardia duodenalis* sequence data. Parasitology. 2007;134(12):1795–821. Epub 06/01. doi: 10.1017/S0031182007003071.

2. Cacciò SM, Beck R, Lalle M, Marinculic A, Pozio E. Multilocus genotyping of *Giardia duodenalis* reveals striking differences between assemblages A and B. Int J Parasitol. 2008;38(13):1523–31.

Supplementary Table 3. The multiple alignments of the SSU rDNA nucleotide fragment sequences of ST1, ST2, ST3, ST5, ST7, and ST10 of *Blastocystis* isolated in this study with reference sequences retrieved from GenBank, represent the position of intra-nucleotide substitutions in *Blastocystis* subtypes isolates.

Isolate/access no				
**ST1**	172	195	247	260
U51151	G	C	C	C
MK801411	A	T	T	G
SC15, SC27, SC29, H15, H23	A	T	T	.

**ST2**	15	171	174	175	177	178	180	181	184	186	188	192	193	194	195	196	197
EU445491	A	A	A	G	A	C	G	A	T	A	C	A	C	T	G	C	T
AB107669	T	G	C	A	G	A	A	G	A	T	T	G	T	G	T	T	C
H8, H27	T	.	.	.	.	.	.	.	.	.	.	.	.	.	.	.	.

**ST3**	164	261
AB107963	A	C
AB107965	.	G
H10, H18, H21	G	.
H24	.	G

**ST5**	176	185	186	187	385
AB107964	G	A	A	T	A
AB107966	.	T	.	.	.
SC80	.	.	.	.	G

**ST10**	169	171	173	175	185	192	196	197	198	199	201	202
KC148207	C	C	A	G	A	C	T	A	G	C	T	C
SC104	G	T	G	C	T	G	C	T	C	T	C	T

ST7	182	193	194	198	201	203	204	209	210	219	227	228	230	231	232	233	234	236	237	238	239
AF408427	T	A	T	A	T	T	A	A	G	A	G	G	T	G	C	C	T	A	G	C	T
AB070991	.	.	.	.	.	.	.	.	.	.	.	.	.	.	.	T	C	G	A	T	.
H19	A	T	A	G	C	C	.	G	A	T	A	A	C	T	.	A	A	.	A	T	C
H29	A	T	A	G	C	C	T	G	A	T	A	A	.	A	T	A	A	G	T	A	C

ST7	241	243	249	250	251	252	253	254	255	256	257	259	269	301	302	306	307	308	309	312	314	321
AF408427	T	T	G	T	T	A	G	G	G	T	A	T	A	A	G	T	T	T	A	C	-	A
AB070991	C	.	.	.	.	.	.	.	.	.	.	.	.	G	.	-	-	-	G	.	-	G
H19	C	C	A	C	.	.	T	T	.	A	G	C	T	.	.	-	-	-	.	G	T	.
H29	.	.	T	.	C	G	T	T	A	.	.	C	T	.	A	-	-	-	.	G	T	.
